# Editorial: the 21st annual *Nucleic Acids Research* Web Server Issue 2023

**DOI:** 10.1093/nar/gkad517

**Published:** 2023-06-13

**Authors:** Dominik Seelow

**Affiliations:** Exploratory Diagnostic Sciences, Berlin Institute of Health, 10117Berlin, Germany; Institute of Medical Genetics and Human Genetics, Charité - Universitätsmedizin Berlin, corporate member of Freie Universität Berlin and Humboldt-Universität zu Berlin, 13353Berlin, Germany

You are reading the 21st edition of the *Nucleic Acids Research* Web Server Issue. The COVID-19 pandemic seems to be over and in many countries, life has returned to normal. However, war is still raging in many parts of the world and there, the situation is far from normal.

For the 2023 Issue, we received 261 proposals (compared to 301 in the last year) and we invited 101 (38%) to submit a manuscript. After peer-review, 81 (31%) manuscripts were eventually accepted.

This year marks my first as editor where I did not co-author any manuscript and did not have any conflict of interests either. I still needed the advice of our senior executive editors Julian Sale and Barry Stoddard when handling some manuscripts – a big thank you to you!

The main work from our side however came from the great team at OUP (especially Martine Bernardes Silva, Rhiannon Meaden, Ajit Minj and the typesetters).

And, of course, from our technical reviewers:

- Oliver Küchler, PhD student, Berliner Institut für Gesundheitsforschung@Charité, Berlin, Germany
- Lennard Ostendorf, MD & PhD, Charité – Universitätsmedizin Berlin, Germany
- Georges Schmartz, PhD student, Universität des Saarlandes, Saarbrücken, Germany
- Adam Simpkin, PhD, University of Liverpool, UK
- Viktoria Wagner, PhD student, Universität des Saarlandes, Saarbrücken, Germany

Oliver from my group has recently joined our team – welcome on board.

As in the previous years, all proposals undergo a rigorous technical review before we invite authors to submit a manuscript. Apart from testing the function of the application, we also evaluate the user-friendliness, the quality of the documentation, the suitability of the licence (it must guarantee free access, also for commercial use) and the ability to analyse user data. We also look for cookies – in the last years, many websites have been built using third-party frameworks and many of them include cookies cross-tracking the users. The latter is prohibited for the Web Server Issue and the use of cookies always requires a consent form or at least a notification. We also do not allow websites to force users to log in, to register, or to enter their email address. The only exception where a registration is permitted (and even encouraged) are web servers handling sensitive human data such as genomes or transcriptomes, but registration must not be mandatory for non-human data. We also encourage web servers to *allow* users to leave their email to be notified when results are available.

The technical review is very time-consuming and it can occasionally take some time before the authors receive the approval or rejection letter. I’d like to thank all of our authors for their patience while we undertake the very rigorous evaluation required for publication in the Web Server issue.

For the next year, we are planning to change the procedures: The proposal upload portal at https://nar.bihealth.de/ will undergo changes:

- In case of classifiers, we will explicitly ask for the publications in which the method was described and validated by peer-review.
- We will also ask for previous publications describing the web server or predecessors. The Web Server Issue only publishes web servers which had not been described in other publications within the preceding two years.
- To better balance the technical reviewing, the upload portal will open on 1 November—we encourage all authors to submit their proposals as soon as possible to avoid an overflow of new submissions within the last days before the deadline.
- Users will be able to see the status of their proposal in the upload portal.

The 2023 Web Server Issue contains the following 81 applications as shown in table on next page.

**Table:** 

Short title	Short description	URL
3D-GNOME 3.0	Analyse 3D genome organization	https://3dgnome.mini.pw.edu.pl/
αCharges	Calculation of partial atomic charges in protein structures	https://alphacharges.ncbr.muni.cz/
Abalign	Multiple sequence alignment for B-cell receptor immune repertoires	http://cao.labshare.cn/abalign/
ACFIS 2.0	Fragment-based drug design	http://chemyang.ccnu.edu.cn/ccb/server/ACFIS2/
AlloReverse	Multiscale analysis of multiple allosteric regulations	http://www.allostery.net/AlloReverse/
AnnotSV 2023	Annotation and ranking of human structural variants	https://www.lbgi.fr/AnnotSV/
antiSMASH 7.0	Secondary/specialized metabolite genome mining	https://antismash.secondarymetabolites.org/
ARMADiLLO	Analysis of antibody mutation probabilities	https://armadillo.dhvi.duke.edu
Breeze 2.0	Visual analysis and comparison of drug-response data	https://breeze.fimm.fi/v2/
CAID	Intrinsic disorder and binding regions in proteins	https://caid.idpcentral.org/submit
CAVE	Analysis and visualization of metabolic pathways	https://cave.biodesign.ac.cn/
ChemMaps.com v2.0	Exploring the environmental chemical universe	https://sandbox.ntp.niehs.nih.gov/chemmaps/
ChroKit	Exploration and multidimensional analysis of heterogeneous genomic data (stand-alone)	https://github.com/ocroci/ChroKit
CRISPRimmunity	Acr prediction for CRISPR/Cas	http://www.microbiome-bigdata.com/CRISPRimmunity
CUPP 2.0	Search for similarities between carbohydrate-processing enzymes.	https://cupp.info/
dbCAN3	Carbohydrate-active enzyme and substrate annotation	https://bcb.unl.edu/dbCAN2/
DDMut	Predicting variant effects on protein stability	https://biosig.lab.uq.edu.au/ddmut
DeepAlloDriver	Prediction of cancer driver mutations	https://mdl.shsmu.edu.cn/DeepAlloDriver
DeepNeo	Prediction of immunogenic neoantigens	https://deepneo.net
DEPICTER2	Prediction of intrinsic disorder	http://biomine.cs.vcu.edu/servers/DEPICTER2/
DIANA-microT 2023	miRNA target prediction	http://www.microrna.gr/microt_webserver/
DIANA-miRPath v4.0	Online miRNA analysis	http://www.microrna.gr/miRPathv4
e-RNA	Prediction and visualization of RNA secondary structures	http://www.e-rna.org
Enrichr-KG	Gene set enrichment analysis	https://maayanlab.cloud/enrichr-kg
FLUXestimator	Study the metabolic fluxome	http://scflux.org/
FunARTS	Identification of fungal bioactive compounds	https://funarts.ziemertlab.com
FuzPred	Prediction of context-dependent protein-protein interactions	https://fuzpred.bio.unipd.it
g:Profiler 2023	Functional enrichment analysis	https://biit.cs.ut.ee/gprofiler
GeneRanger and TargetRanger	Finding differentially expressed genes and proteins	https://generanger.maayanlab.cloud/ https://targetranger.maayanlab.cloud/
Genome Context Viewer version 2	Visual comparison of different genomes	https://gcv.legumeinfo.org/
GenomeFLTR	Removing contaminations from NGS reads	https://genomefltr.tau.ac.il/
GePI	Text-mining for molecular interactions	https://gepi.coling.uni-jena.de/
GPS 6.0	Prediction of kinase-specific phosphorylation sites in proteins	https://gps.biocuckoo.cn
GS-SMD	Molecular dynamics simulations for γ-secretase	https://gs-smd.biomodellab.eu/
Haplogrep 3	Human mitochondrial haplogroups	https://haplogrep.i-med.ac.at
Human AGEs	Human archeogenomics	https://archeogenomics.eu/
IntFOLD7, MultiFOLD and ModFOLDdock	Prediction of protein structures, functions and interactions	https://www.reading.ac.uk/bioinf/
IRSOM2	Prediction of bifunctional RNAs	https://evryrna.ibisc.univ-evry.fr/
KVFinder-web	Detection and characterization of biomolecular cavities	https://kvfinder-web.cnpem.br
The LightDock Server	A macromolecular docking framework	https://server.lightdock.org/
MBROLE3	Functional enrichment analysis of chemical compounds	http://csbg.cnb.csic.es/mbrole3
MicrobiomeAnalyst 2.0	Analysis of microbiome data	http://www.microbiomeanalyst.ca/
miEAA 2023	miRNA enrichment analysis and annotation	https://ccb-compute2.cs.uni-saarland.de/mieaa
Mol*VS	Visualization of volumetric and segmentation imaging data	https://molstarvolseg.ncbr.muni.cz/
MpoxRadar	Monkeypox genomic surveillance dashboard	https://mpoxradar.net
MS^2^PIP	Peptide spectrum predictor for proteomics	https://iomics.ugent.be/ms2pip/
MULocDeep	Protein localization prediction at sub-organellar level	https://www.mu-loc.org/
MyGeneset.info	Sharing and user-created collections of genes	https://mygeneset.info/
NBBC	Explore non-B DNA motifs in cancer	https://kowalski-labapps.dellmed.utexas.edu/NBBC/
nCoVDock2	Prediction of binding modes between COVID-19 targets and potential ligands	https://ncovdock2.schanglab.org.cn
NormSeq	Normalization and differential expression analysis of RNA-seq data	https://arn.ugr.es/normSeq
OnTarget	Design of mini promoters	http://ontarget.cmmt.ubc.ca/
OpenXGR	Enrichment and subnetwork analyses for genes, variants, or genomic regions	http://www.openxgr.com
OrthoVenn3	Exploration of orthologous data across genomes	https://orthovenn3.bioinfotoolkits.net
PAE Viewer	Estimate the reliability of structure predictions generated by Alphafold-Multimer	http://www.subtiwiki.uni-goettingen.de/v4/paeViewerDemo
PanDrugs2	Prioritizing cancer therapies using individual multi-omics data.	https://www.pandrugs.org/
PANGEA	Gene set enrichment analysis for model organisms.	https://www.flyrnai.org/tools/pangea/
PASSer	Prediction of protein allosteric sites	https://passer.smu.edu
PEP-FOLD4	Structure prediction of short peptides considering pH and salt concentration	http://bioserv.rpbs.univ-paris-diderot.fr/services/PEP-FOLD4
Pest Alert Tool	Search for marine non-indigenous species in New Zealand using NGS data	https://pest-alert-tool-prod.azurewebsites.net/
PHASTEST	Identification, annotation and visualization of prophage sequences within bacterial genomes and plasmids	https://phastest.ca
PhD-SNPg	Study the functional impact of short DNA variants	http://snps.biofold.org/phd-snpg
PlasMapper 3.0	Generating and editing plasmid maps	https://plasmapper.ca
PrismNet	Predicting protein RNA interaction	http://prismnetweb.zhanglab.net/
ProAct	Quantification of differential biological process activity	https://netbio.bgu.ac.il/ProAct/
Proksee	Characterization and visualization of bacterial genomes	https://proksee.ca
ResFinderFG v2.0	Search for antibiotic resistance genes	https://cge.food.dtu.dk/services/ResFinderFG/
RNAcanvas	Interactive drawing of nucleic acid structures	https://rnacanvas.app
RNAincoder	Prediction of RNA-associated interactions	https://idrblab.org/rnaincoder/
SEanalysis 2.0	Analysis of super enhancers	http://licpathway.net/SEanalysis
SEPPA-mAb	Spatial epitope prediction of protein antigens for monoclonal antibodies	http://www.badd-cao.net/seppa-mab/
sfkit	Secure and federated genomic data analysis	https://sfkit.org
SH2db	Information system for Src Homology 2 (SH2) domains	http://sh2db.ttk.hu/
SMDB	Interactive exploration of spatial transcriptomics data	https://www.biosino.org/smdb
STellaris	Interactive exploration of spatial transcriptomics data	https://spatial.rhesusbase.com
TCRmodel2	High-resolution modelling of T cell receptor recognition	https://tcrmodel.ibbr.umd.edu.
TransCRISPR	Design optimal single guide RNAs for CRISPR/Cas9	https://transcrispr.igcz.poznan.pl/transcrispr/
tvBOT	Visualization, annotation, and modification of phylogenetic trees.	https://www.chiplot.online/tvbot.html
vissE.cloud	Gene set enrichment analysis	https://www.visse.cloud/
WebQUAST	Evaluation and comparison of genome assemblies	http://cab.cc.spbu.ru/quast/
WebTetrado	Explore quadruplexes in nucleic acid 3D structures	https://webtetrado.cs.put.poznan.pl/

Whilst many of the web servers cover ‘common’ topics such as protein structure predictions or variant annotation and evaluation, there are also some ‘niche’ applications, e.g. for human archeogenomics or biodiversity assessments in marine NGS samples. I hope you enjoy the variety as much as I do.

A very positive development is that more and more web servers use repositories to make their code accessible. Whilst this is not mandatory for the Web Server Issue, we encourage authors to share their code.

Should you wish to submit a manuscript to the 2024 Web Server Issue, please check https://academic.oup.com/nar/pages/submission_webserver for the changes in the procedure. The team at NAR and the scientific community are looking forward to novel and user-friendly web applications.

With this I am coming to an end. My last words go to our referees: reviewing for the Web Server Issue is very challenging because we do have short turn-around times and I know how hard it can be to squeeze a thorough review of a manuscript *and* a web server into our normal work. Thank you very, very much!

